# Assessment of MMP-9, TIMP-1, and COX-2 in normal tissue and in advanced symptomatic and asymptomatic carotid plaques

**DOI:** 10.1186/1477-9560-9-6

**Published:** 2011-04-03

**Authors:** Liz Andréa V Baroncini, Lia S Nakao, Simone G Ramos, Antonio Pazin Filho, Luiz Otávio Murta, Max Ingberman, Cristiane Tefé-Silva, Dalton B Précoma

**Affiliations:** 1 Department of Health and Scienses - Pontifícia Universidade Católica do Paraná, Rua Imaculada Conceição 1155, Curitiba - Paraná - CEP:80215901 - Brazil; 2 Department of Basic Pathology - Universidade Federal do Paraná, Centro Politécnico, Curitiba - Paraná - CEP:80531980 - Brazil; 3 Department of Pathology -Faculdade de Medicina de Ribeirão Preto/USP, Avenida Bandeirantes 3900, Ribeirão Preto - São Paulo - CEP:14049900 - Brazil; 4 Division of Emergency Medicine - Faculdade de Medicina de Ribeirão Preto/USP, Rua Bernardino de Campos 1000, Ribeirão Preto - São Paulo - CEP:14015130 - Brazil; 5 Department of Physics and Math - Faculdade de Filosofia, Ciências e Letras de Ribeirão Preto/USP, Avenida Bandeirantes 3900, Ribeirão Preto - São Paulo - CEP:14049900 - Brazil

## Abstract

**Background:**

Mature carotid plaques are complex structures, and their histological classification is challenging. The carotid plaques of asymptomatic and symptomatic patients could exhibit identical histological components.

**Objectives:**

To investigate whether matrix metalloproteinase 9 (MMP-9), tissue inhibitor of MMP (TIMP), and cyclooxygenase-2 (COX-2) have different expression levels in advanced symptomatic carotid plaques, asymptomatic carotid plaques, and normal tissue.

**Methods:**

Thirty patients admitted for carotid endarterectomy were selected. Each patient was assigned preoperatively to one of two groups: group I consisted of symptomatic patients (n = 16, 12 males, mean age 66.7 ± 6.8 years), and group II consisted of asymptomatic patients (n = 14, 8 males, mean age 67.6 ± 6.81 years). Nine normal carotid arteries were used as control. Tissue specimens were analyzed for fibromuscular, lipid and calcium contents. The expressions of MMP-9, TIMP-1 and COX-2 in each plaque were quantified.

**Results:**

Fifty-eight percent of all carotid plaques were classified as Type VI according to the American Heart Association Committee on Vascular Lesions. The control carotid arteries all were classified as Type III. The median percentage of fibromuscular tissue was significantly greater in group II compared to group I (*p *< 0.05). The median percentage of lipid tissue had a tendency to be greater in group I than in group II (*p *= 0.057). The percentages of calcification were similar among the two groups. MMP-9 protein expression levels were significantly higher in group II and in the control group when compared with group I (p < 0.001). TIMP-1 expression levels were significantly higher in the control group and in group II when compared to group I, with statistical difference between control group and group I (p = 0.010). COX-2 expression levels did not differ among groups. There was no statistical correlation between MMP-9, COX-2, and TIMP-1 levels and fibrous tissue.

**Conclusions:**

MMP-9 and TIMP-1 are present in all stages of atherosclerotic plaque progression, from normal tissue to advanced lesions. When sections of a plaque are analyzed without preselection, MMP-9 concentration is higher in normal tissues and asymptomatic surgical specimens than in symptomatic specimens, and TIMP-1 concentration is higher in normal tissue than in symptomatic specimens.

## Background

The complex structures of mature carotid plaques complicates their histological classification. Patients may exhibit the same carotid plaque histological components whether they are symptomatic or asymptomatic. Fibrotic and calcific plaques could become vulnerable, where as complex plaques with surface defects, hemorrhage, and thrombus could remain silent [1,2]. At present, it is not possible to predict whether a carotid plaque will become symptomatic or when symptoms will occur [3]. The natural history of plaque progression and destabilization is unknown, but it has been suggested that progression of atherosclerosis is a summation of sequential repetitive events resulting in plaque stabilization and plaque destabilization [4]. Carotid atherosclerotic plaque remodeling and an increased risk of symptomatic plaque rupture seem to be partially mediated by matrix metalloproteinases (MMPs). Overexpression of active MMPs contributes to the dissolution of collagenous matrix in the fibrous cap, causing structure to weaken and become brittle and more susceptible to rupture when exposed to hemodynamic stress [5-7]. The expression of 72-kDA (MMP-2) and 92-kDa (MMP-9) gelatinase has been demonstrated within human atherosclerotic lesions and is implicated in plaque rupture and, consequently, in acute ischemic events [8,9]. However, previous reports have failed to detect a positive correlation between MMP-9 and symptomatic plaques. Instead, the level of MMP-9 appears to be related to the age of the patient and the quantity of fibrous tissue in the plaque [5,10-12]. Expression of the tissue inhibitor of MMP (TIMP) is related to plaque stabilization and is increased in calcified areas of carotid plaques [8]. Cyclooxygenase - 2 (COX-2) is involved in MMP production by monocytes [13,14]. We conducted this study to investigate whether MMP-9, TIMP-1, and COX-2 have different expression levels in advanced symptomatic versus asymptomatic carotid plaques removed from surgical patients and in normal tissue in carotid arteries removed from cadavers.

## Methods

### A. Patients

Thirty-six nonconsecutive surgical inpatients admitted for carotid endarterectomy for extracranial high-grade (^3 ^70%) internal carotid artery stenosis were selected for this study. Local ethical committee approval was obtained for the study and for procurement of specimens. Written informed consent was obtained from all patients. Exclusion criteria were: (1) a disorder that could seriously complicate surgery (3 patients); (2) terminal cancer (1 patient); and (3) patient refusal of operation (2 patients). The study was conducted on 30 common or internal carotid artery plaques from the 30 remaining patients (21 males, mean age 68.03 ± 7.3 years). A clinical examination was performed that included a neurological exam to establish the number and duration of ischemic events. The time elapsed between the last symptom and the surgery was recorded for each patient. Before surgery, all patients underwent the following: (1) either cerebral angiography or magnetic resonance angiography with Duplex ultrasound for grading carotid artery stenosis and assessment of the intracranial arterial system; and (2) either computed tomography (CT) or magnetic resonance imaging (MRI) of the brain. The presence or absence of infarction in the corresponding middle cerebral artery territory was noted. Focal cerebral ischemic events were defined as transient ischemic attack (TIA), amaurosis fugax (AF), central retinal artery occlusion, or cerebrovascular accident. Patients were considered to be symptomatic if they had experienced AF, TIA, or stroke ipsilateral to the carotid lesion being studied. Silent infarcts and lacunar symptomatology, diagnosed by a neurologist based on clinical and brain CT and/or MRI located ipsilateral to the stenosis, were also considered symptomatic. Conversely, patients without any history of recent neurologic symptoms or with nonspecific, nonhemispheric symptoms such as dizziness and vertigo were considered asymptomatic. Among asymptomatic patients with stenosis >70%, the indication of surgical therapy depended on comorbidity and vertebro-basilar (in)sufficiency. Each patient was assigned preoperatively to one of two groups on the basis of their symptoms: group I consisted of symptomatic patients (n = 16, 12 males, mean age 66.7 ± 6.8 years), and group II consisted of asymptomatic patients (n = 14, 8 males, mean age 67.6 ± 6.81 years). At the baseline examination, height, weight, body mass index, blood pressure, fasting serum total cholesterol, high density lipoprotein cholesterol, low density lipoprotein cholesterol, triglycerides, fasting plasma glucose, electrocardiograms, and information regarding coronary artery disease, diabetes mellitus, and smoking habits were obtained from each patient. Percentages of carotid diameter reduction, procedural surgical methods, concomitant therapy, age, sex, and risk factors did not differ substantially among the two groups (Table 1). Nine carotid arteries were removed from human adult cadavers that had not been preselected for carotid artery disease. These specimens lacked any macroscopic signs of atherosclerotic plaques and were used as controls.

**Table 1:** Patient baseline characteristics

	Group I (n = 16)	Group II (n = 14)
Age, years	66.7 ± 6.8	67.6 ± 6.8
Sex, M/F	12/4	8/6
Hypertension	10	1
Diabetes Mellitus	2	3
Active smoking	3	3
Hypercholesterolemia	2	1
CAD	4	0
Aspirin	15	10
Statin	5	4
ACE inhibitors	9	8
Ticlopidine	4	1

### B. Tissue specimens and histological analyses

Carotid plaques were obtained immediately after endarterectomy. All surgeries were performed using standard surgical techniques and with minimal manipulation of the specimen. No attempts were made to evaluate the presence and the degree of surface ulceration or thrombus. All plaques were removed en bloc, without fragmentation or significant distortion. After removal, the plaque section to be used for histological analysis was placed in fresh 4% paraformaldehyde solution and partially decalcified overnight for subsequent sectioning. The samples were transected transversely at 3-4 mm and embedded in paraffin. For most of the specimens, five or six blocks were available. Histological analysis was performed based on American Heart Association (AHA) classification system for human atherosclerotic lesions [15,16]. Tissue specimens also were analyzed for lipid, fibrous tissue, and calcium. Results for each tissue component were expressed as a percentage of the total plaque area obtained as described previously [10,11]. Picro sirius red staining of collagen and fat was visualized using polarized light, smooth muscle cells (SMC) were labeled with alpha actin, and calcifications and fat were haematoxylin eosin stained. For tissue characterization a video camera (Leica Microsystems, Bannockburn, IL, US) was applied to a Leica microscope DMR (Leica Microsystems) and attached to a computer. The images were processed using Leica QWin software (Leica Microsystems Image Solutions, Cambridge, UK). Sections to be used for MMP-9, TIMP-1, and COX-2 quantification were snap-frozen in liquid nitrogen and stored at -70°C until processing. The human carotid arteries removed from cadavers received identical treatment.

### C. Immunoblotting

The protein expression levels of MMP-9, TIMP-1, and COX-2 in tissue extracts were analyzed by immunoblotting following SDS-PAGE. Specific regions of each plaque were not preselected for this analysis. Briefly, carotid samples (n = 44) were minced and homogenized in lysis buffer containing PBS (pH 7.2), 0.5% Triton X-100, 0.1% SDS, 0.5% sodium deoxycholate, and protease inhibitor cocktail (Roche) at 4°C. The extracts were centrifuged (5000 rpm, 15 min, 4°C), and supernatants were collected and frozen at

-20°C until analysis. Total protein concentrations in the lysates were determined using the Bradford method. Proteins (10 μg) were separated through a 10% acrylamide/bisacrylamide SDS gel and transferred to a nitrocellulose membrane. After incubation in nonfat milk, membranes were probed with anti-MMP (1:1000), anti-COX-2 (1:250), or anti-TIMP-1 (1: 50) antibodies (Santa Cruz Biotechnology), followed by incubation in secondary antibodies conjugated to horseradish peroxidase (HRP). Reactions were developed using WestPico (Pierce, Rockford, IL, US). As an endogenous control, membranes were probed with anti-β actin (1:5000; Sigma-Aldrich, St. Louis, MO, US) and anti-mouse IgG conjugated to HRP (KLP 1:2000; Kirkegaard and Perry Laboratories, Gaithersburg, MD, US). Densitometry analysis was performed using ImageJ software.

## Statistical analysis

Categorical variables were expressed as percentages, and continuous variables were expressed as means ± SD and medians. The Shapiro-Wilk test was used to evaluate sample normality. For quantitative parameters, the Mann-Whitney nonparametric test was used to compare groups. The Spearman coefficient was used to identify correlations between quantitative variables. Statistical significance was indicated by a value of *p *< 0.05. Analyses were performed using Statistica v. 8.0.

## Results

### A. Histological analysis

Fifty-eight percent of all carotid plaques were classified as Type VI according to the AHA classification system (i.e., complex plaque with possible surface defect, hemorrhage, or thrombus; Table 2). All carotid arteries removed from human cadavers were classified as Type III (i.e., preatheroma with extracellular lipid pools; Table 2). The percentages of lipid, fibromuscular tissue, and calcium were determined for each plaque section (Table 3). The median percentage of fibromuscular tissue was significantly greater in group II (75.9% ± 3.6) than in group I (60.4% ± 5.4; p < 0.05). The median percentage of lipid tissue in group I (32.3% ± 4.7) exhibit a nonsignificant tendency to be greater than group II (19.5 ± 3.2, p = 0.057). The percentage of calcium did not differ significantly between groups (Table 3). The control group had no fibromuscular tissue, lipid tissue, or calcium.

**Table 2:** AHA classification of atherosclerotic lesions across clinical groups

AHA Type	Group I (n = 16)	Group II (n = 14)
Type IV (n)	2	0
Type V (n)	4	2
Type VI (n)	7	11
Type VII (n)	2	0
Type VIII (n)	1	1

**Table 3:** Representation of fibromuscular tissue, lipid, and calcification in plaques across clinical groups (median ± sd)

		Group I (16)	Group II (14)	p
**Histological**	Fibromuscular tissue (%)	60.4 ± 5.4	75.9 ± 3.6	0.047
**Parameters**	Lipid (%)	32.3 ± 4.7	19.5 ± 3.2	0.057
	Calcification (%)	7.3 ± 2	4.5 ± 2.3	0.383

### B. Expression of MMP-9, COX-2, and TIMP-1

MMP-9 expression was significantly elevated in group II and in the control group when compared to group I (p < 0.001; Table 4). COX-2 did not differ significantly across groups. TIMP-1 levels were significantly higher in the control group and in group II than in group I, with statistical difference between control group and group I (p = 0.01; Table 4). No statistical correlation was detected between MMP-9, COX-2, and TIMP-1 levels and fibrous tissue (data not shown).

**Table 4:** MMP-9, COX-2, and TIMP-1 expression (in arbitrary units of band densitometry, normalized by β-actin expression), across the groups

Variable	Group	Mean	Median	SD	p value
MMP-9	GI	127	147	44.3	
	GII	198.9	201.3	11.5	
	Control	182.1	181	25.2	<0.001
COX-2	GI	2.7	2.3	1.6	
	GII	3.3	2.7	1.8	
	Control	2.9	2.1	2.1	0.739
TIMP-1	GI	1.2	0	1.8	
	GII	2.1	2.1	1.8	
	Control	3.6	2.4	2.7	0.039

## Discussion

The carotid plaques examined in the present study were obtained from highly stenotic lesions presumably representing the final stage of plaque development and destabilization. We verified that all surgical specimens had a high grade of complexity. This highlights the challenge of trying to separate vulnerable from stable carotid plaques based only on histological analysis. Lesions considered advanced by their histology may or may not narrow the arterial lumen, may or may not be visible by angiography, and may or may not produce clinical manifestations [15]. The AHA classification for human atherosclerotic lesions considers the presence or absence of different tissue components in plaques but does not account for the proportion of each component. Therefore, the classification of vulnerable or asymptomatic plaques is based on patients' symptoms. When histologic analysis is used to classify plaques as vulnerable or asymptomatic, specific regions inside plaques (e.g., the plaque shoulder) are assessed. Several reports suggest that vulnerability to plaque rupture is a multifocal phenomenon, particularly at the time of acute presentation. However, the imbalanced degradation and synthesis of the extracellular matrix persists in advanced lesions, particularly in plaques with disruption [17]. The mechanisms linking MMP-9 expression within plaques and atherosclerosis still are speculative and maybe explained as an epiphenomenon of the increased number of macrophages within vulnerable plaques [18]. However, plaques from MMP-9 knockout mice are associated with a lower macrophage content compared with those of wild type mice [19], suggesting a primary role of MMP-9 in macrophage migration and activity. Macrophages have a pivotal role in the transition between inflammation and repair; they participate in the wound-healing process via matrix degradation, neovascularization, and recruitment and proliferation of fibroblasts. From a group of 526 symptomatic patients, Redgrave et al. [20] observed a strong correlation between macrophage infiltration and plaque stability. These researches also reported a tendency for plaque inflammation and overall instability to persist with time after TIA but to decrease with time after stroke. The percentage of macrophage area and the percentage of MMP-9 area are markers of plaque instability [21-23]. Kunte et al [24], assessing symptomatic patients with stroke or TIA, found a higher median content of T cells/mm^2 ^and higher median total percentages of macrophage area and MMP-9 area in patients with thromboembolic cerebral ischemia when compared with patients with hemodynamic stroke. They analyzed the tissue specimens in sections with selection of specific regions close to the lipid core in the shoulder region of the plaque. In the present study, we did not assess macrophage, T-lymphocyte infiltration, nor did we select specific sites of extensive inflammation to measure MMP-9, TIMP-1, and COX-2 expressions levels. We verified that MMP-9 is expressed at all stages of atherosclerotic lesions, from normal tissue to advanced plaques. When the plaque was analysed as a whole, rather than specifically in areas of hemorrhage or thrombosis, high concentrations of MMP-9 were detected in normal tissues and in asymptomatic surgical specimens. Peeters et al [23], studying MMP-2, MMP-8, and MMP-9 levels in 804 symptomatic and 174 asymptomatic patients, determined that MMP-levels did not change following stroke except for the normal decrease in macrophage infiltration with time. These investigators suggested that MMPs also can be produced by other cell types such as SMC, which tended to increase with time after stroke, thereby balancing the macrophage-related effects on MMP levels. Furthermore, it has been suggested that MMPs are associated with SMC migration, suggesting that gelatinases play a role in lesion stabilization [25]. In the present study, we classified the patients according to the presence of symptoms and did not differentiate between TIA or stroke or the mechanisms of cerebral ischemia. In addition, we did not quantify SMC. Another important aspect of plaque vulnerability is the plasma concentration of MMP-9. In a study analyzing the MMP-9 activity in asymptomatic patients' plaques, Turu et al. [26] reported that increased plaque activity was correlated with a lower MMP-9 plasma level. This may suggest that circulating MMP-9 levels do not only result from the coexistence of brain ischemic lesions, but in asymptomatic patients, MMP-9 may be also important in atherogenesis. We did not determine the plasma level of MMP-9 in the present study; however, we tried to identify more inflammatory activity by measuring COX-2 expression within plaques. Cipollone et al [8] suggested that synthesis of COX-2 and prostaglandin E synthase (PGES) by activated macrophages is associated with acute ischemic syndromes, possibly through MMP-induced plaque rupture. Higher expression levels of COX-2, PGES, MMP-2, and MMP-9 were detected in specimens obtained from the carotid lesions of patients with recent TIA or stroke compared with specimens obtained from asymptomatic patients. Specimens were preselected for plaque ulceration and intraplaque hemorrhage before analysis. Because we did not preselect specimens for suspicious areas, and instead analyzed all sections of the plaque, we could not detect any correlation between COX-2, MMP-9, and TIMP-1expression and patients' symptoms. Finally, immunocytochemical studies suggest that nondiseased human arteries and experimental animal arteries uniformly express MMP-2 and the inhibitory TIMP-1 and TIMP-2 [27]. The present study corroborates these findings because we observed a high concentration of MMP-9 and TIMP-1 in normal human arteries. Future studies should focus on the genetic variants of MMPs and features of the atherosclerotic plaques that could lend insight into the mechanism by which MMPs, ultimately, influence plaque structure and vulnerability [28].

## Conclusions

MMP-9 and TIMP-1 are present in all stages of atherosclerotic plaque progression, from normal tissue to advanced lesions. When no preselected region of a plaque is analyzed, the MMP-9 concentration is higher in normal tissues and in asymptomatic surgical specimens, suggesting that MMP-9 is part of the atherogenesis process and is not specifically a factor in acute disruption events. TIMP-1 expression levels were higher in normal tissues and in asymptomatic specimens than in symptomatic specimens. COX-2 concentrations did not differ among groups. The quantification of macrophages and SMC should be a focus in future studies.

## Competing interests

The authors declare that they have no competing interests.

## Authors' contributions

LAVB, DBP and APF designing and wrote the study

LSN, SGR, CTS, MI and LOMJ performed the histological and immunoblotting analysis.

All authors read and approve the final manuscript.
